# Mesenchymal stem cells promote lymphangiogenic properties of lymphatic endothelial cells

**DOI:** 10.1111/jcmm.13590

**Published:** 2018-05-11

**Authors:** Jan W. Robering, Annika Weigand, Romy Pfuhlmann, Raymund E. Horch, Justus P. Beier, Anja M. Boos

**Affiliations:** ^1^ Department of Plastic and Hand Surgery Laboratory of Tissue Engineering and Regenerative Medicine University Hospital of Erlangen Friedrich‐Alexander University of Erlangen‐Nürnberg (FAU) Erlangen Germany; ^2^ Present address: Department of Plastic Surgery, Hand and Burn Surgery University Hospital RWTH Aachen Aachen Germany

**Keywords:** growth factors, lymphangiogenesis, lymphatic endothelial cells, mesenchymal stem cells

## Abstract

Lymphatic metastasis is one of the main prognostic factors concerning long‐term survival of cancer patients. In this regard, the molecular mechanisms of lymphangiogenesis are still rarely explored. Also, the interactions between stem cells and lymphatic endothelial cells (LEC) in humans have not been well examined. Therefore, the main objective of this study was to assess the interactions between mesenchymal stem cells (MSC) and LEC using in vitro angiogenesis assays. Juvenile LEC were stimulated with VEGF‐C, bFGF, MSC‐conditioned medium (MSC‐CM) or by co‐culture with MSC. LEC proliferation was assessed using a MTT assay. Migration of the cells was determined with a wound healing assay and a transmigration assay. To measure the formation of lymphatic sprouts, LEC spheroids were embedded in collagen or fibrin gels. The LEC's capacity to form capillary‐like structures was assessed by a tube formation assay on Matrigel^®^. The proliferation, migration and tube formation of LEC could be significantly enhanced by MSC‐CM and by co‐culture with MSC. The effect of stimulation with MSC‐CM was stronger compared to stimulation with the growth factors VEGF‐C and bFGF in proliferation and transmigration assays. Sprouting was stimulated by VEGF‐C, bFGF and by MSC‐CM. With this study, we demonstrate the potent stimulating effect of the MSC secretome on proliferation, migration and tube formation of LEC. This indicates an important role of MSC in lymphangiogenesis in pathological as well as physiological processes.

## 1 INTRODUCTION

In recent years, regenerative medicine and tissue engineering using stem cells has become a prime interest of research all over the world. The identification and characterization of stem cells provide the basis for ongoing preclinical and clinical trials of organ and tissue regeneration using healthy adult stem cells.^1^

There are different types of cell sources for transplantation and tissue engineering purposes. Mesenchymal stem cells (MSC) are able to differentiate into cell types of different germ layers.^2^ They demonstrate a lower developmental potential and shorter lifespan than pluripotent embryonic stem cells and are not known to facilitate the spontaneous formation of tumours. Furthermore, they also exhibit immunosuppressive properties upon transplantation.^3^ It has already been described that MSC positively influence angiogenesis of blood vessels and the revascularization of ischemic tissue through the secretion of blood endothelial cell (BEC) stimulating factors.^4^ MSC also contribute to the formation of tumour blood vessels via integration as atypical vascular endothelial growth factor A (VEGF‐A) secreting cells.^5^ In contrast to the well‐characterized relationship between BEC and MSC, however, little is known about lymphatic endothelial cell (LEC)‐MSC interaction.

The survival rate associated with a certain type of cancer is mainly determined by the tumour cell's ability to form distant metastases. Cancer cells can disseminate from the primary tumour site via haematogenic and lymphatic routes.^6^ Starting from the sentinel lymph node, they spread to other lymph nodes and distant organs.^7^ Lymphatic vessels participate in tumour metastasis providing channels for tumour cells to leave lymph nodes^8^ and play a complex role in metastatic tumour spread.^9^ While the molecular mechanisms of lymphangiogenesis are still rarely explored, some of the involved growth factors and molecular signalling pathways have already been discovered.^10^ One of the most studied group of pro‐lymphangiogenic growth factors are VEGFs.^11^ VEGFs are highly specific mitogens for vascular endothelial cells. They induce endothelial cell proliferation, promote cell migration and inhibit apoptosis. It is known so far that VEGF‐C is the main lymphangiogenic growth factor in both physiological und pathological settings.^12^ After processing, VEGF‐C develops a higher affinity for VEGFR‐3, which is exclusively expressed on LEC.^13^ The expression of VEGF‐C first occurs during embryogenesis, but remains high in adult lymph nodes.^14^ The VEGF‐C/VEGFR‐3 signalling pathway is essential for tumour‐associated lymphangiogenesis.^15^ VEGFs not only influence lymphangiogenesis directly but also interact with other factors both directly and indirectly. One of these factors is the basic fibroblast growth factor (bFGF), which is also known as FGF2. Together with VEGF‐C, it synergistically promotes lymphangiogenesis in the tumour microenvironment.^12^ Furthermore, bFGF directly induces LEC proliferation and migration via activation of FGFR‐1. Another important lymphangiogenic growth factor is hepatocyte growth factor (HGF), which also promotes proliferation, migration and tube formation of LEC via its receptor HGF‐R.^16^ HGF directly affects lymphangiogenesis and is not dependent on VEGFR3 activation.^17^

In order to make further advances in the fields of tissue engineering and regenerative medicine as well as to address questions related to the lymphatic spread of tumour cells, a better understanding of the underlying mechanisms of lymphangiogenesis and the interactions between LEC, other cells, and in particular stem cells is needed. In contrast to the well‐characterized interactions between MSC and BEC, to the best of our knowledge, the paracrine interactions between MSC and LEC have not been studied in detail using primary human cells until now.^18^, ^19^ Therefore, the aim of this study was to evaluate the in vitro interactions of LEC and MSC as a basis for further lymphangiogenesis and metastasis research.

## 2 MATERIALS AND METHODS

### 2.1 Cell culture

Human dermal LEC derived from juvenile foreskin (HDLEC) were purchased from PromoCell GmbH (Heidelberg, Germany) and cultured in endothelial cell growth medium MV (ECGM MV, PromoCell) with the corresponding supplement mix (see C‐22020 PromoCell). LEC in passages 6 and 7 were used for all experiments.

Human MSC derived from bone marrow (hMSC‐BM) were purchased from PromoCell and cultured in MSC growth medium (MSC medium, MSC‐GM, PromoCell) with the corresponding supplement mix (see C‐28010 PromoCell). MSC in passages 6 and 7 were used for all experiments.

Culture medium was changed 3 times a week, and the cells were passaged 1:3 after reaching a confluence of 80%. All cells were cultured at 37°C in an atmosphere of 5% CO_2_.

### 2.2 MSC‐conditioned medium

MSC were seeded in T75 flasks (Greiner Bio‐One, Frickenhausen, Germany). After the MSC reached confluence, the medium was removed and the cells were washed once with PBS (Biochrom GmbH, Berlin, Germany). The MSC were incubated for 48 hours with 10 mL of endothelial cell basal medium (ECBM, PromoCell) containing 0.5% FBS (foetal bovine serum, FBS superior; Biochrom GmbH). After 48 hours, the culture medium was collected and used for experiments.

### 2.3 Proliferation assay

LEC were seeded in 96‐well plates at a density of 2.5 × 10^3^ cells/well in 100 μL ECGM. The cells were given 4 hours to adhere. To characterize the effect of MSC‐secreted growth factors on LEC proliferation and to determine the optimal MSC‐CM concentration, culture medium was replaced either with 0.5 % FBS ECBM containing growth factors for stimulation or with MSC‐CM. The responses of LEC to different concentrations of MSC‐CM (10%, 30%, 50%, 70% and 100%) were examined. For evaluation of growth factor effects on LEC proliferation, the medium was removed and the cells were treated with 100 μL ECBM supplemented with 0.5% FBS and 200 ng/mL recombinant human VEGF‐C (Peprotech, Rocky Hill, NJ, USA), 100 ng/mL recombinant human bFGF (Peprotech), a combination of both growth factors or 100% MSC‐CM. A positive control was performed with 100 ng/mL PMA (Axxora by Enzo Life Sciences, Farmingdale, New York, USA), as negative control ECBM supplemented with 0.5% FBS was used. Cells were stimulated for 24, 48 and 72 hours at 37°C under 5% CO_2_.

Cell proliferation activity was measured using Cell Proliferation Kit I according to the manufacturer's instructions (MTT, Roche Diagnostics GmbH, Mannheim, Germany). Ten microlitres of the MTT labelling reagent (final concentration 0.5 mg/mL) was added to each well and incubated for 4 hours. Afterwards, 100 μL solubilization solution was added into each well and incubated overnight. The absorbance was measured using a microplate reader (Multiskan™ GO, Thermo Fisher Scientific, Waltham, MA, USA) at a wavelength of 595 nm with a reference wavelength of 690 nm.

### 2.4 Migration assay

LEC migration was assessed with a scratch assay and a transmigration (Boyden chamber) assay. For the scratch assay, 4 x 10^5^ cells/well were seeded in a 6‐well plate. As soon as the cells reached confluence, a lesion was generated in a standardized fashion using a 1000 μL pipette tip and the cells were cultivated with 2 mL ECBM containing 0.5% FBS as a negative control; ECBM containing 0.5 % FBS and 400 ng/mL VEGF‐C in combination with 200 ng/mL bFGF, 200 ng/mL PMA or 100% MSC‐CM for 12 and 24 hours at 37°C under 5% CO_2_. Images of the lesion were captured with an inverted microscope (Olympus IX81, Olympus Corporation, Tokyo, Japan) at 10‐fold magnification in four random fields after 12 and 24 hours. Live cell imaging was performed with a Olympus cellVivo incubation system. Migration of the cells was analysed by measurement of the uncovered area (Photoshop CS6 Extended, Adobe Systems GmbH, Munich, Germany).

Transmigration of the LEC was assessed using tissue culture‐treated transwell chambers with a diameter of 6.5 mm and 8.0 μm pore size membrane (Corning Inc., Corning, NY, USA). Transwell membranes were coated with 0.2% gelatine (Carl Roth, Karlsruhe, Germany) for 1 hour at 37°C under 5% CO_2_. 1 x 10^5^ LEC suspended in 100 μL ECBM containing 0.5 % FBS were seeded in the upper chambers. The lower chambers were filled with 600 μL ECBM supplemented with 0.5% FBS and 400 ng/mL recombinant human VEGF‐C in combination with 200 ng/mL recombinant human bFGF. To demonstrate the stimulating effect of the MSC secretome on LEC migration, 100% MSC‐CM or 7.5 x 10^5^ MSC suspended in 600 μL ECBM supplemented with 0.5% FBS was filled in the lower transwell chamber. 200 ng/mL PMA served as positive control. ECBM supplemented with 0.5% FBS was used as negative control. After incubation for 16 hours at 37°C in a 5% CO_2_ atmosphere, the cells on the upper surface of the membrane were removed using a cotton swab and the cells on the lower surface of the filter were fixed with 100% ice‐cold methanol and stained with DAPI (Roche Diagnostics GmbH). Images of the transmigrated LEC were captured at 10‐fold magnification in 4 random fields, and the migrated cells were counted with the Olympus cellSens imaging software (version 1.12).

### 2.5 Tube formation assay

The formation of three‐dimensional capillary‐like structures was examined by performing a Matrigel^®^‐based tube formation assay. Each well of a μ‐slide (ibidi GmbH, Martinsried, Germany) was filled with 10 μL of growth factor‐reduced Matrigel^®^ (Corning Inc.), which was allowed to polymerize for 30 minutes at 37°C. The LEC were labelled with cell tracker CM‐DiI (Life Technologies by Thermo Fisher Scientific) and the MSC with CellTrace Oregon Green 488 (Life Technologies by Thermo Fisher Scientific). The LEC were seeded in the wells at a density of 6 × 10^3^ cells/well and cultured with 45 μL ECBM supplemented with 0.5% FBS and 800 ng/mL recombinant human VEGF‐C (Peprotech) in combination with 400 ng/mL recombinant human bFGF (Peprotech) or 100% MSC‐CM. A positive control was performed with 400 ng/mL PMA (Axxora), as negative control ECBM supplemented with 0.5% FBS was used.

Furthermore, the LEC were co‐cultivated with 1,000 or 2,000 MSC in 45 μL ECBM containing 0.5% FBS. After 16‐hour incubation, the tube formation images were captured at 4‐fold magnification using the Olympus IX83. The total tube length, number of tubes, area covered by tubes and branching points were quantified using the WimTube analysis software (Wimasis GmbH, Munich, Germany).

For the immunofluorescent staining of LEC in the tube formation assay, adherent MSC were labelled with Cyto ID (4 minutes in CytoID Solution, CytoID‐Kit by Enzo Life Sciences). Either 5,000 unlabeled LEC, 2,000 labelled MSC or a combination of both were seeded on Matrigel^®^ in μ‐slides as described above. For the podoplanin staining, cells were fixed with 4% phosphate‐buffered formaldehyde for 15 minutes and washed with PBS. After removal of PBS, blocking solution was added (60 minutes room temperature, 5% FCS in PBS). Afterwards, 10 μg/mL Alexa 594 anti‐human Podoplanin AB (BioLegend, San Diego, California, USA) were added per well and incubated overnight at 4°C. Wells were washed 3 times with PBS. Stained cells were washed with PBS overnight at 4°C.

### 2.6 Sprouting assay

LEC spheroids were prepared in hanging drops. The LEC were suspended in 25 μL drops of ECGM containing 0.24% carboxymethylcellulose (Sigma‐Aldrich, St. Louis, MO, USA). Each Drop harboured 500 cells and was pipetted on a square Petri dish and cultivated upside down at 37°C under 5% CO_2_. After 48 hours, spheroids were harvested and embedded in collagen or fibrin gels as described by Korff et al^20^ A collagen stock solution was prepared prior to use by mixing an acidic collagen extract of rat tails (equilibrated to 2 mg/mL, 4°C; 8 vol.) with medium 199 (Sigma‐Aldrich; 1 vol.) and 0.1 N NaOH (approx. 1 vol.) to adjust the pH to 7.4. This stock solution (0.5 mL) was mixed with 0.5 mL ECBM with 20% FBS and 0.5% carboxymethylcellulose to prevent sedimentation of the spheroids prior to polymerization of the collagen gel. For the fibrin gels, 500 spheroids/gel were suspended in a solution of 25 μL medium 199, 725 μL ECBM and 1.5 U/mL thrombin and then mixed with 250 μL fibrinogen (6 mg/mL, Baxter Deutschland GmbH, Unterschleißheim, Germany).

The spheroid‐containing gels were quickly transferred into preheated 24‐well plates and allowed to polymerize for 30 minutes. 100 μL ECBM supplemented with 0.5% FBS and 400 ng/mL recombinant human VEGF‐C (Peprotech) in combination with 200 ng/mL recombinant human bFGF (Peprotech) or 100% MSC‐CM was pipetted on top of the gels. 200 ng/mL PMA served as positive control. ECBM supplemented with 0.5% FBS was used as a negative control.

The gels were incubated at 37°C in a 5% CO_2_ atmosphere. After 24 hours, images were taken with an inverted microscope (Olympus IX81) and sprouting was analysed with the Olympus cellSens imaging software. The sprout length was measured in 4 pictures of every group (triplicates) of three independent experiments and the number of sprouts was counted by ImageJ software. Data analysis was performed with Microsoft Excel 2010 (Microsoft Cooperation, Redmond, WA, USA).

### 2.7 ELISA analyses of different growth factors

To measure the concentration of growth factors relevant for lymphangiogenesis in the MSC‐CM, ELISAs for VEGF‐C, VEGF‐D, HGF and bFGF were performed. MSC‐CM was prepared as previously described, aliquoted and frozen at ‐80°C for later use. All assays were performed according to the manufacturer's instructions. VEGF‐C, VEGF‐D and HGF ELISA kits were manufactured by DLdevelop (Wuxi, Jiangsu, China) and the bFGF ELISA Kit by BioLegend (San Diego, California, USA). Each sample was measured in duplicate. MSC‐CM was generated with three different MSC isolations from different patients n = 3, Donor 1: 64 y/male/Caucasian; Donor 2: 36 y/female/Caucasian; Donor 3: 91 y/female/Caucasian.

### 2.8 Statistical analysis

Data from all experiments are displayed as the mean of all independent experiments ± standard deviation (SD). The statistical analysis was performed with SPSS 21 by IBM. As negative control, ECBM with 0.5% FBS was used. Levene′s test was performed to test for homogeneity of variance. The test was always non‐significant, except for the scratch, transmigration and sprouting assay. One‐way ANOVA was performed for comparing multiple samples. Tukey′s test was conducted as a *post hoc* test. Student's *t*‐test was performed for pairwise comparisons (ELISA measurements). Differences were considered statistically significant at *P* ≤ .05 and highly significant at *P* ≤ .01.

## 3 RESULTS

### 3.1 Effect of different concentrations of MSC‐CM on LEC

**Figure 1:**
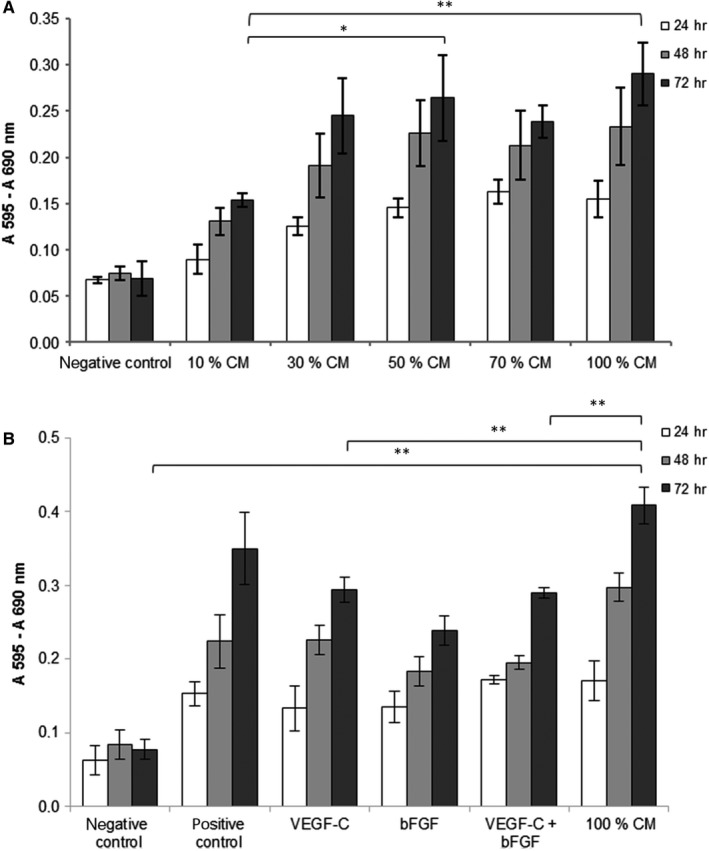
Effect of MSC‐conditioned medium (CM) on LEC proliferation in the MTT assay. Bar graphs show a comparison of viability represented in absorbance (*y*‐axis) of LEC treated with different stimulations (*x*‐axis) at 24‐72 h. (n = 3, triplicate, *P ≤ .05, **P ≤ .01; only the significances of 72 h are marked) (A) Cells were treated with different dilutions of MSC‐CM. (B) Cells were treated with 100% MSC‐CM, VEGF‐C, bFGF and the combination of both growth factors. PMA served as positive control, basal medium supplemented with 0.5% FCS as negative control

After 72 hours, stimulation with every dilution of MSC‐CM resulted in a highly significant increase of LEC proliferation in comparison to the negative control (Figure 1A). At 72 hours, cultivation with 30%, 50% and 70% MSC‐CM did not show any significant differences between the concentrations. However, 10% MSC‐CM stimulated cell proliferation significantly less compared to higher concentrations like 50% or 100%. Therefore, 100% MSC‐CM was used in all following experiments.

### 3.2 MSC‐CM stimulated LEC proliferation to a higher extent than the combination of VEGF‐C and bFGF

After 24, 48 and 72 hours, stimulation with VEGF‐C and bFGF as well as 100% CM resulted in significant higher LEC proliferation in comparison with the negative control (Figure 1B). Stimulation with PMA (positive control) or MSC‐CM resulted in a significantly higher proliferation compared to VEGF‐C, bFGF or the combination of both. After 48 hours, LEC proliferation was highest in MSC‐CM. At 72 hours, proliferation was significantly increased by MSC‐CM in comparison with the negative control and to the growth factors VEGF‐C and bFGF. LEC proliferation was significantly increased by bFGF or VEGF‐C stimulation alone and in combination with both compared to the negative control.

### 3.3 MSC‐CM stimulated LEC migration to the same extent as VEGF‐C and bFGF

**Figure 2:**
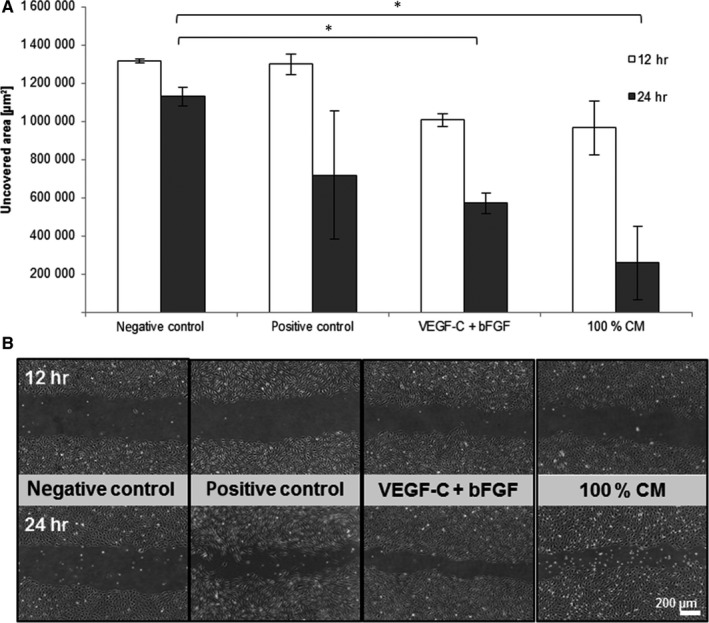
Effect of MSC‐CM on LEC migration in the scratch assay. LEC were treated with VEGF‐C combined with bFGF and 100% MSC‐CM. PMA served as positive control, basal medium supplemented with 0.5% FCS as negative control. (A) Bar graphs show a comparison of migratory activity represented in uncovered scratch area (*y*‐axis) of LEC treated with different stimulations (*x*‐axis) at 12 and 24 h. (n = 3, duplicates, *P ≤ .05; only the significances of 24 h are marked) (B) Representative images of LEC migration

In the scratch assay, LEC migration was significantly increased by VEGF‐C + bFGF and MSC‐CM compared to the negative control group after 12 hours (Figure 2A,B). MSC‐CM induced LEC migration to the same extent as the combination of growth factors VEGF‐C and bFGF and was significantly increased after 12 hours compared to the positive control PMA. After 24 hours, migration was significantly increased in all groups compared to negative control.

### 3.4 MSC‐CM and indirect co‐culture of LEC and MSC as well as MSC‐CM stimulated LEC transmigration

**Figure 3:**
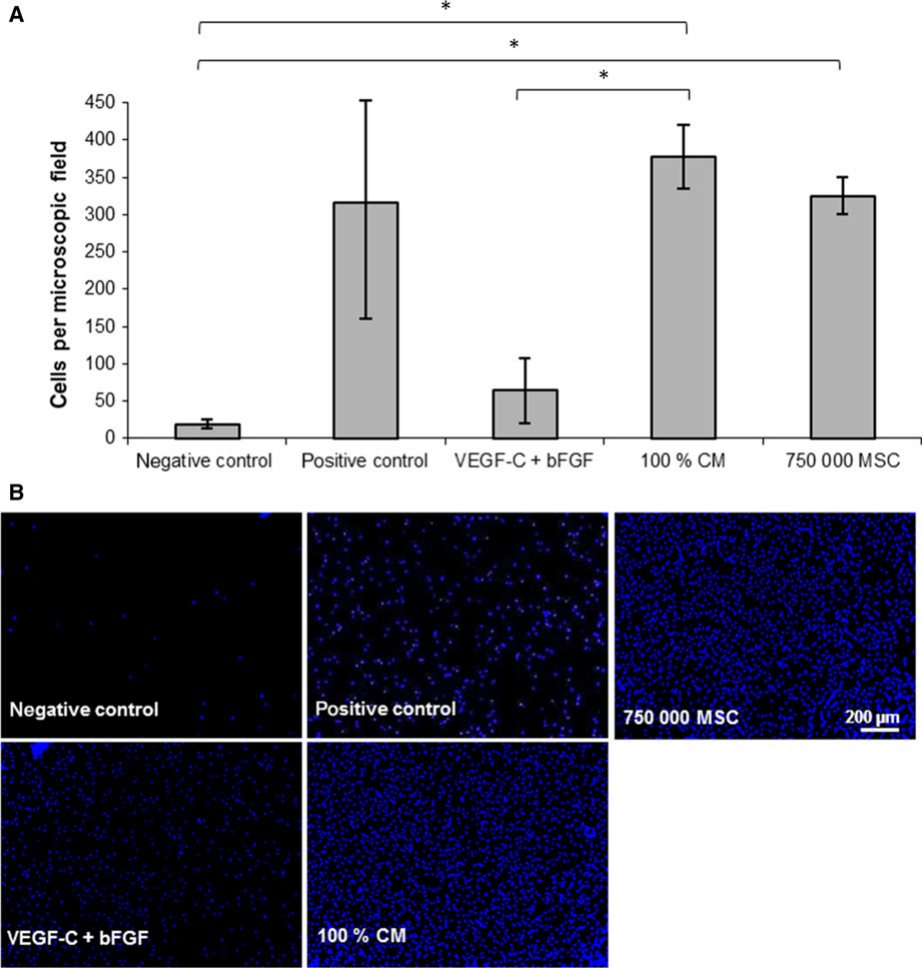
Effect of MSC‐CM on LEC transmigration in a modified Boyden chamber assay. The lower compartment of the transwell chamber was loaded with VEGF‐C combined with bFGF, 100% MSC‐CM or 750,000 MSC. PMA served as positive control, basal medium supplemented with 0.5% FCS as negative control. (A) Bar graphs show a comparison of migratory activity represented in the average number of transmigrated LEC per field of vision (*y*‐axis) of cells treated with different stimulations (*x*‐axis). (n = 3, duplicates, *P ≤ .05) (B) Representative images of LEC transmigration

Cultivation of LEC with MSC‐CM resulted in a significantly increased number of transmigrated cells in comparison with the negative control and to the growth factors (Figure 3A,B).

Cultivating MSC in the lower compartment of the well plate significantly enhanced LEC transmigration compared to the negative control. A cell count of 750,000 MSC was necessary to achieve a transmigration rate similar to that of MSC‐CM stimulation (Figure 3A,B).

### 3.5 Formation of capillary‐like structures was stimulated by MSC‐CM and co‐cultivation with MSC

**Figure 4:**
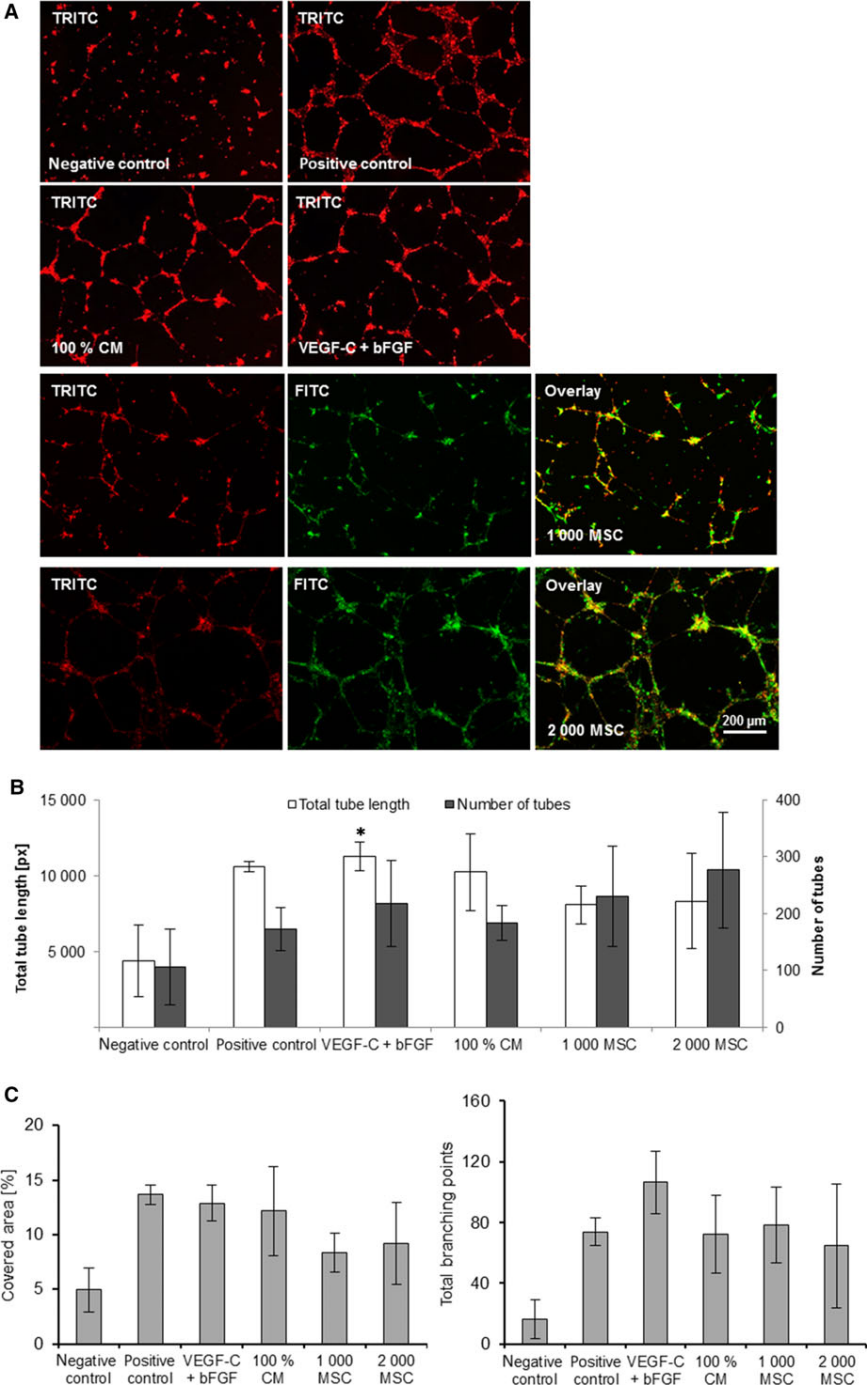
Effect of MSC‐CM on LEC tube formation. LEC were treated with VEGF‐C combined with bFGF, 100% MSC‐CM or co‐cultured with 1,000 or 2,000 MSC. PMA served as positive control, basal medium supplemented with 0.5% FCS as negative control. (n = 3, singles, *P ≤ .05 compared to negative control) (A) Representative images of LEC tube formation. LEC were labelled in red, MSC in green. (B) Bar graphs show the total tube length (left *y*‐axis) and the number of tubes (right *y*‐axis) of LEC treated with different stimulations (*x*‐axis). (C) Bar graphs show the tube‐covered area (left) and the number of branching points (right) of LEC treated with different stimulations (*x*‐axis)

In comparison with the negative control, the formation of vessel‐like structures could be enhanced by adding MSC‐CM or through co‐cultivation with MSC (Figure 4A‐C). In the co‐culture group, capillary‐like structures were formed both by LEC and MSC (Figure 4A). In each group, longer tubes were measured compared to the negative control. Concerning the total tube length, there was no difference between MSC‐CM, co‐cultivation with MSC, the positive control or the combined growth factors VEGF‐C and bFGF (Figure 4B). The covered area was slightly increased in the positive control, the growth factor group and the MSC‐CM group compared to the negative control. In the MSC group with 2,000 cells, a significantly larger area was covered while there was no difference between the group with 1000 MSC and the negative control (Figure 4C). In every group, except the MSC groups, more total branching points were measured compared to the negative control without differences between the groups (Figure 4C). MSC alone are also able to form tubes but to a lesser extend than LEC + MSC together. In LEC‐MSC co‐cultures, both cell types contributed to tube formation demonstrated by podoplanin staining (Figure S1).

### 3.6 Sprouting of LEC in fibrin gels was equally induced by stimulation with MSC‐CM or the combination of VEGF‐C and bFGF

**Figure 5:**
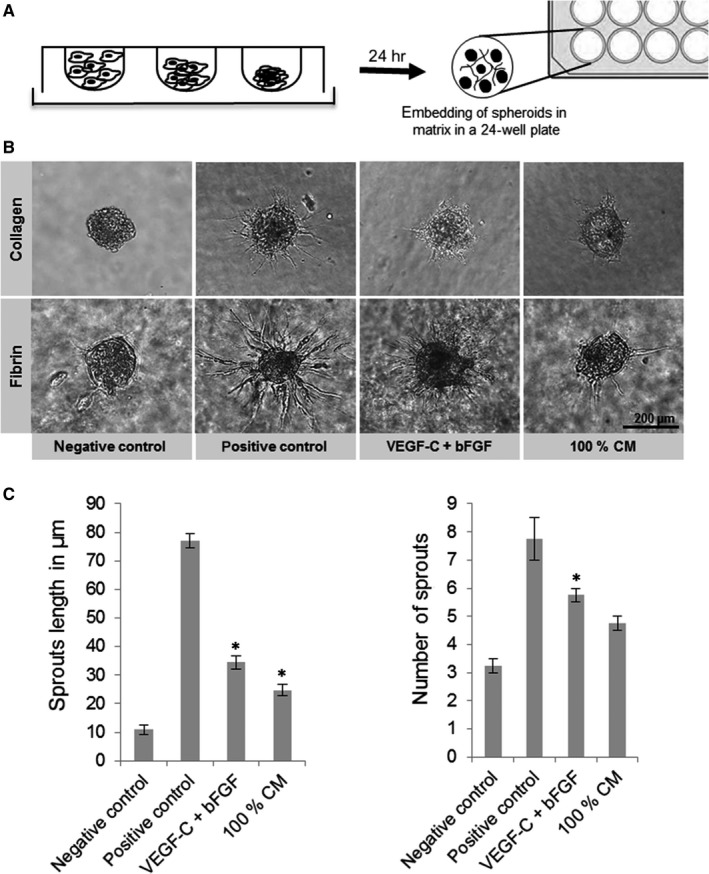
Effect of MSC‐CM on LEC sprouting. LEC spheroids were embedded in collagen or fibrin gels and treated with VEGF‐C combined with bFGF and 100% MSC‐CM. PMA served as positive control, basal medium supplemented with 0.5% FCS as negative control. (A) Spheroids were grown in hanging drop culture. (B) Representative images of spheroids treated with different stimulations after 24 h. (C) Sprout length and number of spheroids treated with different stimulations (*x*‐axis) were quantified and displayed in bar graphs. (n = 2, duplicates, *P ≤ .05 compared to negative control)

Sprouting of LEC spheroids embedded in collagen and fibrin gels could be enhanced by stimulation with the positive control (Figure 5). In terms of VEGF‐C + bFGF, LEC spheroid sprouting was enhanced in the fibrin gels compared to the collagen gels. In both gel types, MSC‐CM stimulated less LEC sprouting than PMA. MSC‐CM stimulated LEC sprouting in fibrin gels to a similar extent as a combination of VEGF‐C and bFGF (Figure 5C).

### 3.7 Concentration of VEGF‐C, HGF and bFGF in the MSC‐CM

**Figure 6:**
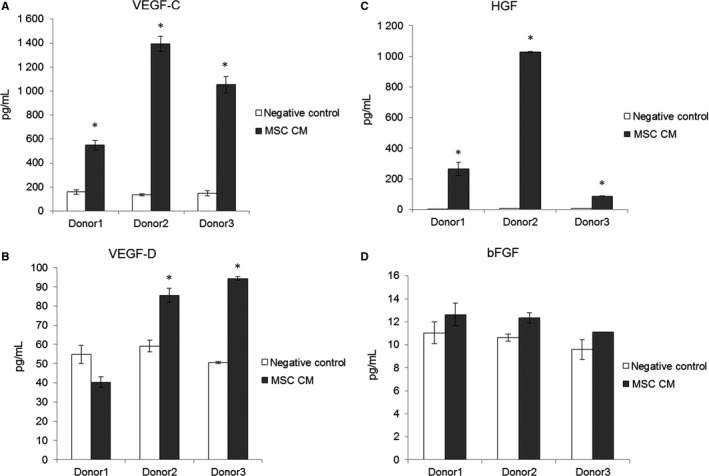
Concentration of different growth factors in MSC‐CM. ELISA analyses of MSC‐CM and basal medium as negative control for VEGF‐C, VEGF‐D, HGF and bFGF are displayed in bar graphs. CM was generated with MSC obtained from 3 different donors. (2 replicates, *P ≤ .05 compared to negative control)

To determine the concentration of growth factors in the MSC‐CM, ELISAs for VEGF‐C, VEGF‐D, HGF and bFGF were performed. VEGF‐C and HGF could be measured in a significantly higher concentration in the MSC‐CM compared to the negative control. High concentrations of VEGF‐C could be measured by ELISA in the MSC‐CM of all patients. The growth factor concentration in the MSC‐CM depends on the donor and differs between single patients. VEGF‐D could be detected in the CM of MSC obtained from two of three donors (Figure 6). The bFGF concentration was nearly equal in control medium and MSC‐CM.

## 4 DISCUSSION

Although it has been shown that MSC positively influence angiogenesis, the interactions between human MSC and LEC and their role in lymphangiogenesis and lymphatic metastasis have not been studied in detail. With this study, we demonstrate the positive influence of MSC and MSC‐CM on LEC proliferation, migration and tube formation, which are important processes during the lymphangiogenic cascade. These mechanisms are mediated through the secretion of pro‐lymphangiogenic factors like VEGF‐C and bFGF acting on the corresponding receptors VEGF‐R3 and FGF‐R3 expressed by LEC.^14^, ^21^, ^22^

It is well‐known that MSC contribute to the formation of new blood vessels. This effect is based on a combination of the direct and indirect influences of MSC on BEC. On the one hand, they secrete several factors directly implicated in angiogenesis such as VEGF‐A, angiopoietin‐1 and bFGF.^23^, ^24^ On the other hand, MSC secrete cytokines such as interleukin‐6, which induce endothelin‐1 production in cancer cells and thereby enhance endothelial cell recruitment and activation in an indirect manner.^25^ Their contribution to lymphangiogenesis has not been investigated in detail yet, especially compared to blood vessel angiogenesis, but it can be assumed that cytokines secreted by MSC (e.g. VEGF, angiopoietin‐2, bFGF and HGF) play a crucial role.^26^ Moreover, MSC can contribute to lymphangiogenesis by transdifferentiation into endothelial cells and incorporation into the vessel wall.^27^, ^28^

The present results indicate that the lymphangiogenic effect of MSC‐CM on proliferation and migration is more effective than stimulation with added growth factors. These results are in line with studies on murine adipose‐derived stem cells (ADSCs) and human LEC by other groups. For instance, human LEC were treated with murine ADSC‐CM and the factors secreted by ADSCs induced LEC proliferation, migration and tube formation more potently than recombinant human VEGF‐C.^29^ ADSCs show characteristics comparable to MSC and secrete multiple (lymph)angiogenic growth factors, such as VEGF, HGF, bFGF, IGF, interleukins 6, 7, 8 and 11, the epidermal growth factor (EGF), the platelet‐derived growth factor (PDGF) and the transforming growth factor beta (TGF‐β).^30^, ^31^ These growth factors are secreted in bioactive levels, whereby VEGF‐C is the main (lymph)angiogenic factor and plays a central role in the paracrine effects of ADSCs.^32^, ^33^ Silencing HGF reduces the ability of ADSCs to promote endothelial cell proliferation and inhibits the proangiogenic effects of HGF in vitro.^34^

In this study, MSC‐CM stimulated migration, proliferation and tube formation of LEC similar compared to added recombinant growth factors or even to a higher extent (concerning the proliferation or transmigration). One explanation for our results could be a synergistic interplay between multiple lymphangiogenic growth factors in the MSC‐CM like HGF or VEGF‐C shown by our ELISA analyses. We could detect VEGF‐C in the MSC‐CM by ELISA. However, it will be the aim of further studies to determine the amount of the active and inactive form of VEGF‐C in the MSC‐CM. In the current study, we focused on the main lymphangiogenic factors VEGF‐C and bFGF.

Further explanations of the high stimulation of MSC‐CM compared to the growth factor groups could be the presence of other lymphangiogenic factors such as angiopoietins, HGF and IGF with lymphangiogenic effects.^35^ Recently it could be shown that HGF and IGF can have a direct effect on lymphangiogenesis.^16^, ^17^ Also chemokines such as CXCL‐12 positively influence lymphangiogenesis and stimulate lymphatic cancer metastasis.^36^, ^37^ Stimulation with VEGF‐C increased expression of the corresponding receptor CXCR4. This effect is based on the VEGF‐C‐mediated activation of HIF1α. Therefore, the CXCL12‐CXCR4 signalling pathway acts independently of the lymphatic VEGF‐C receptor VEGFR‐3, which opens up the possibility of new therapeutic options.^38^, ^39^

A detailed analysis of the MSC‐CM is necessary to identify additional components of the MSC secretome influencing the lymphangiogenic cascade and to gain deeper insights into lymphangiogenesis.

Besides, the secretion of growth factors by stem cells themselves, modulation of the MSCs secretory properties by stimulation with various growth factors could further enhance the MSC effect on lymphangiogenesis. Yan et al showed that short‐term stimulation of ADSCs with VEGF‐C resulted in increased expression of VEGF‐A, VEGF‐C and Prox‐1 in vitro and was associated with a significantly increased lymphangiogenic response.^40^ Furthermore, stimulation of ADSCs with VEGF‐C increased their proliferation and survival after in vivo implantation and induced the expression of podoplanin. Thus, for possible applications of MSC for therapeutic purposes, it would be thinkable to stimulate these cells in advance with recombinant growth factors to induce a higher pro‐lymphangiogenic effect.

Because of their tissue‐like mechanical properties and immunologic integrity, we used fibrin gels for the sprouting assay.^41^ Fibrin matrices for implantations can be autologously harvested from the graft‐recipient themselves and allow the adaptation of the polymerization and degradation rate by varying the concentration of aprotinin.^42^, ^43^ As second hydrogel, we chose collagen because of its abundancy in mammalian tissue^44^ and its successful and common usage in other angiogenesis studies.^45^, ^46^

Compared to all other assays in the present study, the MSC‐CM effect on LEC sprouting was not as high as expected. Although it is believed that the process of lymphangiogenesis is composed of several single steps (invasion, capillary organization, tubular branching, network formation, maturation), the precise mechanisms are still not fully understood. In contrast to the blood vascular endothelium, which is in direct contact with the basement membrane components, lymphatic capillaries lack a basal lamina.^47^ As a result, LEC have to penetrate an interstitial collagen barrier in the extracellular matrix (ECM), for example by matrix metalloproteinase 2 (MMP2) which supports migration and vessel branching of LEC.^48^ Thus, poor sprouting may be because of the inability of LEC to secrete proteins like MMPs. Furthermore, diffusion of MSC‐CM to the LEC spheroids could be impeded by the gel matrix. Another explanation could be a possible dilution of the CM within the gel matrix. In future studies, it would be interesting to analyse the effect of a more concentrated CM.

The results from our in vitro experiments will provide the basis for the in vivo part to follow. To do this, the lymphangiogenic effect of MSC‐secreted factors should be evaluated in the rat arteriovenous (AV) loop model.^49^ This model will subsequently be used for lymphangiogenesis, anti‐lymphangiogenesis and metastasis research in future in vivo studies.^50^, ^51^

## 5 CONCLUSION

In the present study, the interaction between lymphatic endothelial cells (LEC) and mesenchymal stem cells (MSC) was evaluated using several in vitro angiogenesis assays. This study demonstrates the positive influence of a conditioned medium of primary human MSC on the lymphangiogenic response of primary human LEC. The lymphangiogenic growth factors secreted by the MSC enhanced proliferation and transmigration of LEC to a higher extent than the combination of VEGF‐C and bFGF. In the scratch assay, the stimulative effect was similar to the combination of the growth factors VEGF‐C and bFGF, but higher compared to the negative control. Understanding the mechanisms of lymphangiogenesis and the role of the involved growth factors could help to gain deeper insights into the mechanisms of lymphangiogenesis in pathological processes as well as lymphatic metastasis. Furthermore, understanding the mechanism behind the MSC's stimulating effect on endothelial cells is a crucial requirement for the transition of novel MSC‐based therapies from bench to bedside.

## CONFLICT OF INTEREST

All authors state that there is no conflict of interest.

## Supporting information


* *

* *

** **
