# Supplementary figures and images for: Mesenchymal stem cells promote lymphangiogenic properties of lymphatic endothelial cells
Source: J Cell Mol Med. 2018 May 11;22(8):3740–50. doi: 10.1111/jcmm.13590 (PMC6050462; doi:10.1111/jcmm.13590)

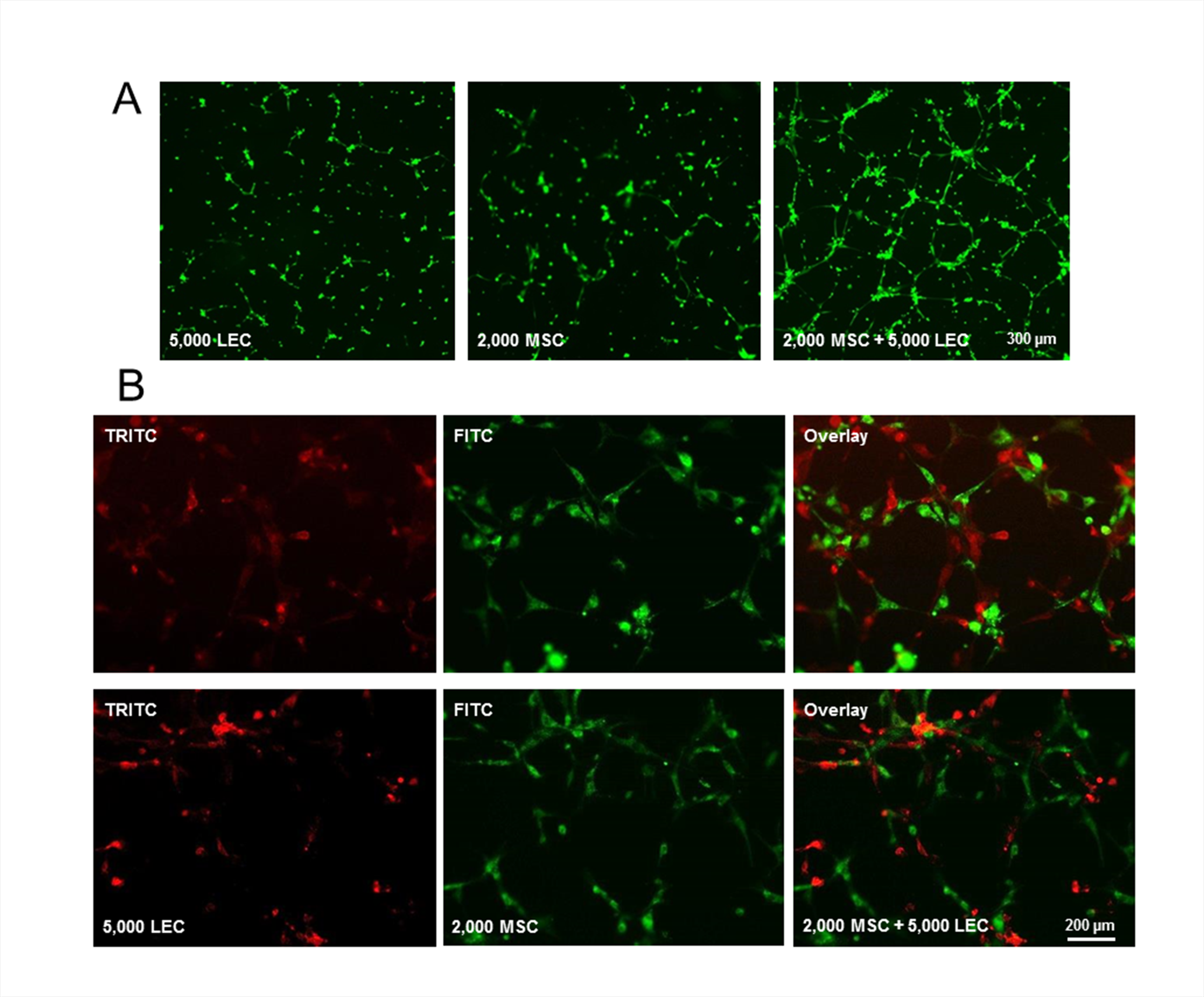

Supplement: Supplementary file 1 [file JCMM-22-3740-s001.tif]
